# UPLC-MS/MS Profiling, Antioxidant, α-Glucosidase Inhibitory, Cholinesterase Inhibitory, and Cardiovascular Protection Potentials of Jialing 20 (*Morus multicaulis* Perr.) Mulberry Branch Extract

**DOI:** 10.3390/foods10112659

**Published:** 2021-11-02

**Authors:** Wei Xiang, Zhining Xia, Li Xu

**Affiliations:** 1 School of Pharmaceutical Sciences, Chongqing University, Chongqing 401331, China; xiangwel@foxmail.com; 2 State Key Laboratory of Silkworm Genome Biology, Key Laboratory of Sericultural Biology and Genetic Breeding, Ministry of Agriculture and Rural Affairs, College of Sericulture, Textile and Biomass Sciences, Southwest University, Chongqing 400715, China

**Keywords:** mulberry branch, UPLC-MS, antioxidant, α-glucosidase, cholinesterase, cardiovascular

## Abstract

As a by-product in the sericulture industry, mulberry branches are not currently utilized effectively. Jialing 20 is an artificial triploids mulberry that widely cultivated in southwest China. In this study, the chemical composition of the Jialing 20 mulberry branch extract (MBE) was first analyzed by UPLC-MS/MS, and 42 components, including alkaloids, flavonoids, and coumarins, were obtained. Then, the antioxidant activities, hypoglycemic effect, Alzheimer’s disease inhibition, and cardiovascular protection of MBE were also evaluated in vitro. The IC_50_ values for the scavenging DPPH and ABTS radicals were, respectively, 31.23 ± 0.57 μg/mL and 8.88 ± 0.36 μg/mL (IC_50_ values of positive Vc were, respectively, 4.41 ± 0.19 μg/mL and 8.79 ± 0.41 μg/mL). The IC_50_ value for inhibiting α-glucosidase was 1.90 ± 0.05 μg/mL (IC_50_ value of positive acarbose was 0.03 μg/mL). The IC_50_ values for inhibiting acetylcholinesterase and butyrylcholinesterase were, respectively, 179.47 ± 0.38 μg/mL and 101.82 ± 3.37 μg/mL (IC_50_ values of positive berberine were, respectively, 1.27 ± 0.03 μg/mL and 57.41 ± 0.21 μg/mL). MBE (10 μg/mL and 40 μg/mL) significantly increased the survival rate of oxidized low-density lipoprotein- (ox-LDL) induced human umbilical vein endothelial cells (HUVECs) and significantly decreased the intracellular reactive oxygen species. These results suggest that the extracts of Jialing 20 mulberry branches could be used as a functional food additive.

## 1. Introduction

Mulberry is a common cash crop in Asia [1]. Mulberry fruits are usually consumed as fresh fruit and the raw material for the production of delicious juices and jams. Mulberry leaves are used to feed domestic silkworms or are made into mulberry tea. However, mulberry branches, which need to be cut regularly every year, are underutilized [2]. Mulberry branches, also known as Ramulus mori (RM), are included in the Chinese Pharmacopoeia as a herbal beverage, and mulberry branch extract (MBE) has been approved as a food source by the Chinese and Korean governments [3,4], indicating that MBE is safe for consumption. Mulberry branches are rich in active components such as flavonoids [5], alkaloids [1], and polysaccharides [6], which have various activities such as antioxidation [7] and hypoglycemic effects [8]. However, the chemical composition and biological activities of MBE of different varieties of mulberry trees vary greatly [7,9,10]. Jialing 20 is an artificially bred triploid mulberry variety and is widely cultivated in southwest China because its leaf and branch yields are significantly better than those of other varieties [11]. In our previous study, 10 components, including mulberroside A, were isolated and identified from MBE of Jialing 20, and the content of mulberroside A in Jialing 20 was found to be significantly higher than that in other cultivars. Mulberroside A is the main bioactive component in mulberry branches [12] and can ameliorate diabetic endotoxemia [13] and protect against hepatic damage [14,15]. However, the active components or biological activities of MBE of Jialing 20 have not been comprehensively explored.

Age-related diseases, including diabetes, Alzheimer’s disease, and cardiovascular diseases, threaten the health of middle-aged and elderly people [16]. Diabetes is a chronic metabolic disease that is characterized by hyperglycemia, and more than 90% diabetic patients have Type 2 Diabetes Mellitus (T2DM) [17]. α-Glucosidase inhibitors are widely used in the treatment of type 2 diabetes [18]. Alzheimer’s disease (AD) is a common degenerative disease of the central nervous system that is characterized by memory and cognitive dysfunction and the decreased ability to perform daily living activities [19]. Cholinesterase inhibitors are currently the main drugs used to relieve AD symptoms [20]. Atherosclerosis (AS) is a common cardiovascular disease, and its development is closely related to the endothelial cell damage mechanism [21]. Oxidized low-density lipoprotein (ox-LDL) can cause vascular endothelial cell damage and dysfunction through various pathways and can thus promote the development of atherosclerosis [22]. ox-LDL-induced vascular endothelial cell damage is a common mechanism for cardiovascular diseases [23]. In the elderly population, several diseases often occur simultaneously, and the occurrence and development process of these diseases is accompanied by oxidative stress and free radical damage [24,25]. Recent studies have shown that adding antioxidants to the body system can prevent and treat the diseases caused by free radicals and the related oxidative stress [26,27]. Therefore, antioxidation and free radical scavenging are important ways to prevent and treat these diseases. Functional food ingredients are substances that are intended to produce a positive effect on health beyond basic nutrition [28]. It is important to search for food ingredient that have the ability to prevent and treat these diseases.

LC-MS is an important technique in natural product research that is widely applied in phytochemical composition analysis [29]. In this study, the small-molecular-weight components in MBE of Jialing 20 were firstly isolated and analyzed by means of UPLC-MS/MS. In addition, in vitro antioxidation activities, the inhibition of α-glucosidase and cholinesterase, and protection against ox-LDL-induced human umbilical vein endothelial cells (HUVECs) damage, were explored. The current study provides a theoretical basis for the effective utilization of the mulberry branches of Jialing 20 in functional foods for hypoglycemia treatment, Alzheimer’s disease prevention, and cardiovascular protection.

## 2. Materials and Methods

### 2.1. Sample Preparation and Extraction

Jialing 20 mulberry branches were cut in the winter from the mulberry garden of Southwest University (29°49′18″ N latitude, 106°24′51″ E longitude), dried at 50 °C using a DHG Series Heating and Drying Oven (Keelrein, Shanghai, China) and crushed using a High-Speed Multi-Purpase Disintegerator 500A (Chencan, Yongkang, China), and then extracted for 30 min by adding methanol at a material-to-liquid ratio of 1:10 (*w*/*v*) with the aid of ultrasound (700 w, 30 min) at room temperature. The extraction step was repeated four times. The extract was evaporated at 50 °C using a rotary evaporator Rotavapor R-100 (Büchi, Flawil, Switzerland) and was then freeze-dried using a vacuum freeze dryer Lab-1C-50E (Biocool, Beijing, China) to obtain mulberry branch extract (MBE).

### 2.2. Chemicals

Acetonitrile, 2,2-Diphenyl-1-picrylhydrazyl (DPPH), 2,2′-Azino-bis(3-ethylbenzothiazoline-6-sulfonic acid) diammonium salt (ABTS), 4-Nitrophenyl α-D-glucopyranoside (*p*NPG), 5,5′-Dithiobis-(2-nitrobenzoic acid) (DTNB), Acetylcholinesterase (AChE), Butyrylcholinesterase (BChE), Acetylthiocholine iodide (ATC), and *S*-Butyrylthiocholine iodide were obtained from Sigma-Aldrich (Shanghai, China). α-Glucosidase was obtained from Solarbio (Beijing, China). Vitamin C (Vc) was obtained from Keshi (Chengdu, China).

### 2.3. UPLC-MS/MS Analysis

#### 2.3.1. Chromatographic Conditions

A Nexera LC-30A liquid chromatography system (Shimadzu, Kyoto, Japan) equipped with a Waters ACQUITY UPLC BEH C18 (2.1 × 100 mm, 1.7 μm particle size) column was used under the following conditions: 30 °C, an injection volume of 2 μL, mobile phase A of 0.1% formic acid–water, and mobile phase B of 0.1% formic acid–acetonitrile. The elution procedure was set as: 0–3.5 min, 5–15% B; 3.5–6 min, 30% B; 6–12 min, 30–70% B; 12–12.5 min, 70% B; 12.5–18 min, 70–100% B; 18–25 min, and 100% B.

#### 2.3.2. Mass Spectrometry Conditions

A TripleTOFTM 5600+ MS system (AB Sciex, Framingham, MA, USA) with an electrospray ion source and an Analyst TF 1.7.1 workstation (AB Sciex, Framingham, MA, USA) was used. The primary and secondary mass spectrometry data acquisitions were performed by using TOF-MS-Product Ion-IDA with a scan range of 50 to 1500 *m/z*. The mass spectrometry parameters were set as follows: declustering voltage of 100 eV or −100 eV and focusing voltage of 10 eV or −10 eV; secondary bombardment energy of 40 eV or −40 eV; collision energy spread (CES) of 20 V; and spray voltage of 5500 V (positive ion mode) or −4000 V (negative ion mode).

#### 2.3.3. Mass Spectrometry Data Processing

The data collected by mass spectrometry were first imported into Progenesis QI software (Waters, USA, Milford, MA, USA). Then, corresponding charge forms were selected for peak matching, alignment, and extraction. Then, the obtained results were matched with the secondary mass spectrometry database for screening and identification based on the literature.

### 2.4. Antioxidant Activity

#### 2.4.1. Scavenging of DPPH Radicals

The scavenging capacity of DPPH radicals was determined according to the previous method [30], with minor modifications. Then, 100 μL of 200 μM DPPH solution was dissolved in ethanol, which was then mixed with the same volume of different concentrations of MBE in a 96-well microtiter plate. After the reaction proceeded at room temperature in the dark for 30 min, the absorbance of the mixture was measured at 520 nm three times using an imark microplate reader (Bio-Rad, Hercules, CA, USA), and water-soluble vitamin C was used as a positive control. The DPPH radical scavenging rate was calculated as:

$$D=\frac{A0-A1}{A0}\times 100\%$$

where *D* is DPPH radical scavenging rate (%); *A*0 is the absorbance of the negative control; and *A*1 is the absorbance of the sample. The semi-inhibitory concentration (IC_50_) of DPPH radicals is calculated according to the derivation of the logarithm of the substrate concentration with respect to inhibition rate.

#### 2.4.2. Scavenging of ABTS Radicals

The scavenging capacity of ABTS radicals was determined according to the previous method [31], with minor modifications. First, 7 mM ABTS was mixed with 2.45 mM potassium persulfate and was incubated in the dark for 12 h. Then, the mixture was diluted to a concentration with an absorbance of 0.700 ± 0.05 at 734 nm. Then, 20 μL of the sample was mixed with 180 μL of the radical solution for a 6 min reaction in the dark. Finally, the absorbances were measured at 734 nm three times using an imark microplate reader (Bio-Rad, Hercules, CA, USA), while vitamin C was used as a positive control. The ABTS radical scavenging rate was calculated as:

$$A=\frac{A0-A1}{A0}\times 100\%$$

where *A* is the ABTS radical scavenging rate (%); *A*0 is the absorbance of the negative control; and *A*1 is the absorbance of the sample.

### 2.5. Inhibition of α-Glucosidase Activity

The inhibition of α-glucosidase activity was determined according to the previous method [32]. An amount of 50 μL of each sample was to be tested, and 50 μL of α-glucosidase (0.5 U/mL) and 50 μL of phosphate buffer (67 mM, pH 6.8) were mixed thoroughly and were kept at 37 °C for 15 min. Then, 50 μL of 6 mM *p*NPG was added, and the reaction proceeded at 37 °C for 15 min. Then, the reaction was terminated by adding 60 μL of 1 M Na2CO3. Three replicates were set for each group. Then, 200 μL of the solution was added in a 96-well plate, and the absorbance value was measured at 405 nm with an imark microplate reader (Bio-Rad, Hercules, CA, USA) in triplicate (*n* = 3). The inhibition rate was calculated as:

$$\alpha -Glucosidase\;Inibition\;\left(\%\right)=\left(1-\frac{A1-A2}{A0-A2}\right)\times 100\%$$

where *A*1 is the absorbance value of each sample; *A*2 is the absorbance value of each sample without the enzyme; and *A*0 is the absorbance value of the group without sample.

### 2.6. Inhibition of Cholinesterase Activity

The inhibition of cholinesterase activity was determined according to the previous method [33], with minor modifications. First, 40 μL of PBS (pH 8.0, 100 mM), 20 μL of DTNB (2 mM), 10 μL of AChE (0.75 U/mL), and 10 μL of samples of different concentrations were mixed followed by the rapid addition of 20 μL of ATC (2 mM) to initiate the reaction. After 2 min, the reaction solution was analyzed with an imark microplate reader (Bio-Rad, Hercules, CA, USA) at 405 nm. The inhibition reaction was determined three times, and the inhibition rate was calculated as:

$$Cholinesterase\;Inhibition\;\left(\%\right)=\left(1-\frac{A1-A2}{A0-A2}\right)\times 100\%$$

where *A*1 is the absorbance value of each sample; *A*2 is the absorbance value of each sample without enzyme; and *A*0 is the absorbance value of the group without sample.

### 2.7. Protection of MBE against ox-LDL-Induced HUVECs Damage

#### 2.7.1. Cell Culture

Human umbilical vein endothelial cells (HUVECs) were purchased from Shanghai Institutes for Biological Sciences, Chinese Academy of Sciences, and were cultured in Dulbecco’s modified Eagle medium (DMEM, Giboc, Waltham, MA, USA) containing 10% fetal bovine serum (FBS, Giboc, Waltham, MA, USA) and 1% penicillin–streptomycin double antibody at 37°C in an incubator (Forma 3111, Thermo Scientific, Waltham, MA, USA) containing 5% CO_2_.

#### 2.7.2. Cytotoxicity

HUVECs in logarithmic growth phase were inoculated overnight in 96-well plates at a concentration of 5 × 10^3^ cells/well and were then treated with different concentrations of samples for 24 h. Six replicates were arranged for each group. Then, 10 μL of CCK-8 solution (CellorLab, Hangzhou, China) was added to each well and was incubated for 1 h in an incubator. The absorbances were measured at 450 nm with an enzyme-labeled instrument to calculate cell viability (*n* = 6).

#### 2.7.3. Cell Vitality

Cell vitality was determined according to the previous method [21], with minor modifications. First, the HUVECs were inoculated overnight in 96-well plates at a concentration of 5 × 10^3^ cells/well and were then treated with different concentrations of samples under the exposure condition of 80 μg/mL ox-LDL (Yiyuanbiotech, Guangzhou, China) for 24 h. Then, 10 μL of CCK-8 solution was added to each well and was incubated for 1 h in an incubator. Six replicates were arranged for each group. The absorbances were measured at 450 nm with an enzyme-labeled instrument to calculate cell viability (*n* = 6).

#### 2.7.4. Determination of Intracellular Reactive Oxygen Species (ROS)

Intracellular ROS was determined with Dihydroethidium (DHE). HUVECs in logarithmic growth phase were first inoculated for 12 h in 6-well plates at a concentration of 1 × 10^5^ cells/well and were then treated for 24 h according to the method in Section 2.7.3. After 10 μM DHE (Beyotime Biotechnology, Shanghai, China) was added, the reaction proceeded for 60 min at 37 °C. Then, the treated HUVECs were rinsed with a blank medium 3 times and were observed with an inverted fluorescence microscope IX73 (Olympus Corporation, Tokyo, Japan) under an excitation wavelength of 518 nm and an emission wavelength of 610 nm.

## 3. Results and Discussion

### 3.1. UPLC-MS/MS Fingerprints

**Figure 1:**
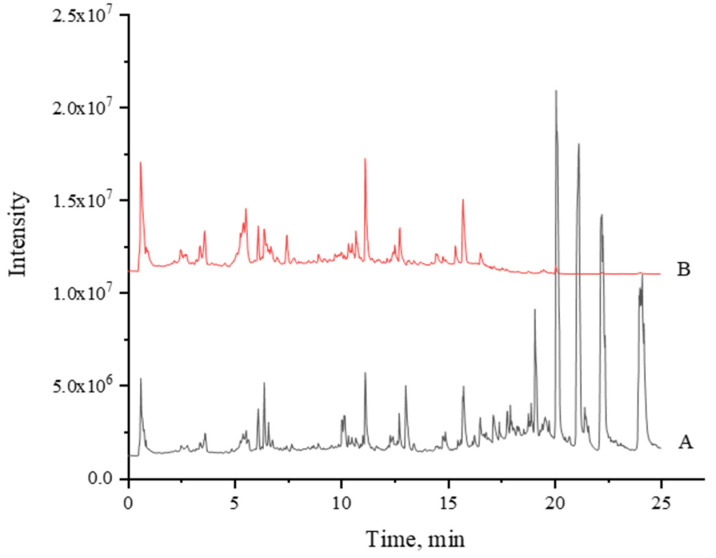
Total ion chromatogram of UPLC-MS of MBE. (**A**), Positive; (**B**), Negative.

The UPLC-MS fingerprint of MBE is shown in Figure 1. In addition to some interference signals from the fatty acids, more signals of weakly polar components in positive ion mode were acquired. More signals of moderately polar components were obtained in negative ion mode. Through signal processing, the mass spectra of 14,284 positive ions and 14,130 negative ions were obtained. The signal extraction and analysis results indicated that the main components of MBE were flavonoids and polyphenols. These components could be subdivided into stilbene, coumarin, phenylpropanoid compounds, alkaloids, and organic acids. Due to the large interference in the signals of the positive ions, the data obtained in negative ion mode are generally used in the LC-MS analysis of natural products. Therefore, in this study, besides the signals of positive ions, the signals of negative ions were mainly taken into account for data analysis. The resolved compounds are shown in Table 1 In total, 42 obvious compound signals were obtained.

Among the compounds identified by LC-MS/MS, four components, including scopolin, reported in our previous study were also found in this study. Oxyresveratrol (Oxr) is commonly found in mulberry branches, including those from Jialing 20 [15,37]. In summary, 37 new components in MBE of Jialing 20 were found, thus enriching knowledge about the chemical composition of the Jialing 20 mulberry branch.

A preliminary comparison of the mass spectral response intensity indicated that the components with higher contents in MBE might be oxyresveratrol and isoprenoid flavonoids (Kuwanon G). These compounds have also been reported to possess antibacterial [38,39] and tyrosinase inhibitory activities [40,41], suggesting that MBE might be used as antibacterial agents and whitening products.

The application of LC-MS/MS technology when studying mulberry mainly focuses on the composition analysis of mulberry leaves [42,43] and mulberry fruits [44,45], but it is seldom applied in the analysis of the main components in mulberry branches. The compounds identified by UPLC-MS/MS of MBE were quite different from the results of mulberry leaves and mulberry in references [42,43,44,45]. The rapid identification of the main components of MBE by LC-MS/MS can improve our understanding of its chemical composition and can provide theoretical guidance for the development and utilization of its chemical components.

### 3.2. Antioxidant Activity

**Figure 2:**
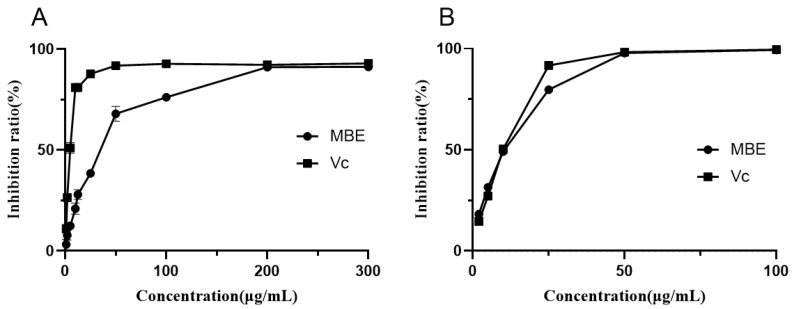
Free radical scavenging activities of MBE. (**A**), DPPH radical; (**B**), ABTS radical.

Free radicals are closely related to the progression of diabetes, neurodegenerative diseases, and atherosclerosis. The scavenging assay of DPPH and ABTS free radicals is a common method to evaluate the free radical scavenging activity in vitro. Figure 2 shows the ability of MBE to scavenge the free radicals DPPH and ABTS. MBE exhibited concentration-dependent DPPH free radical scavenging activity in the concentration range of 0 to 200 μg/mL. The IC_50_ value of MBE was 31.23 ± 0.57 μg/mL, and the IC_50_ value of positive Vc was 4.41 ± 0.19 μg/mL. In the concentration range of 0 to 50 μg/mL, the ability of MBE to scavenge ABTS free radicals was positively correlated with MBE concentration. The IC_50_ value of MBE was 8.88 ± 0.36 μg/mL, and the IC_50_ value of positive Vc was 8.79 ± 0.41 μg/mL. The ABTS scavenging ability of the sample was close to that of positive Vc, but the DPPH scavenging ability of the sample was different from that of positive Vc. The DPPH radical scavenging assay displayed the ability of the sample to transfer electrons or hydrogen atoms, whereas the ABTS radical scavenging assay demonstrated the hydrogen supply ability and chain-breaking ability of the sample [46]. The above results indicated that compared to Vc, MBE was more dominated by the hydrogen supply ability and chain-breaking ability rather than the ability to transfer electrons or hydrogen atoms.

The DPPH radical scavenging activity of the extracts from the same parts of different species of mulberry (*Morus alba* L. and *Morus nigra* L.) differed significantly and was positively correlated with the total content of polyphenols and flavonoids in the extracts, indicating that phenolics might be the main antioxidant components in different Morus extracts [9]. The ethanolic extracts of mulberry branches showed significant antioxidant activities, indicating that oxyresveratrol (Oxr) might be the most effective antioxidant component [47]. Oxr showed a good ability to scavenge DPPH and ABTS radicals. The possible high content of Oxr in MBE of Jialing 20 might be responsible for its better antioxidant activity.

### 3.3. Inhibition of α-Glucosidase Activity

**Figure 3:**
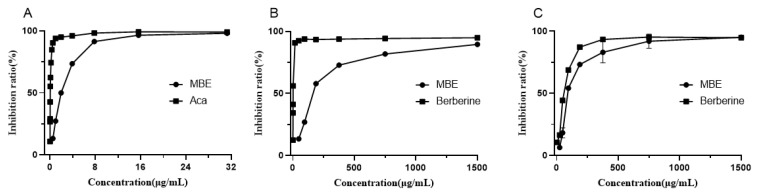
Enzyme inhibitory activity of MBE. (**A**), α-glucosidase; (**B**), AChE; (**C**), BChE.

As drugs for the effective control of T2DM, α-Glucosidase inhibitors have relatively few side effects because they do not need to be absorbed into the blood and can function directly in the intestine. These α-glucosidase inhibitor drugs are currently commercially available, such as acarbose and voglibose. We measured the activity of MBE in inhibiting α-glucosidase with acarbose as a positive control (Figure 3A). When the sample concentration increased from 0 to 8 μg/mL, the inhibition activity of MBE on α-glucosidase increased significantly. When the sample concentration further increased from 8 to 16 μg/mL, its inhibition ability increased slowly. When the sample concentration further increased above 16 μg/mL, its inhibition ability mostly remained stable. Its half-inhibitory concentration was 1.90 ± 0.05 μg/mL (the half-inhibitory concentration of acarbose was 0.03 μg/mL).

In the previous study on α-glucosidase inhibitory components in mulberry (*Morus alba* L.) with molecular docking, three types of potential small-molecule α-glucosidase inhibitory components were identified: alkaloids (1-deoxynojirimycin, DNJ), flavonols (kaempferol, quercetin and rutin), and isoprenoid flavonoids (morin, sanggenon C and kuwanon G) [48]. The LC-MS/MS results showed that DNJ and Kuwanon G in the MBE of Jialing 20 might be the material basis for its inhibition effect on α-glucosidase activity.

Since DNJ is highly polar and is difficult to retain in the C18 column, the LC-MS/MS analysis failed to separate it from other compounds. In addition, it has no UV absorption. This present study determined the DNJ content to be 0.65% in MBE with the reported FMOC-CL method [32]. The IC_50_ value of DNJ was 1.15 ± 0.13 μg/mL, which was similar to that of MBE. However, the content of DNJ was low, indicating that other α-glucosidase inhibitory components with stronger activity than DNJ existed in MBE. The result was consistent with previous results [32].

### 3.4. Inhibition of Cholinesterase Activities

Acetylcholinesterase and butyrylcholinesterase inhibitors can slow down the hydrolysis of acetylcholine and can increase the acetylcholine (Ach) level in the synaptic cleft, thus delaying the development of symptoms in Alzheimer’s patients [49]. Cholinesterase inhibitors such as donepezil and galantamine have been successfully marketed as clinical agents to alleviate the symptoms of Alzheimer’s disease. Berberine, a natural product with cholinesterase inhibitory activity, is often used as a positive control in the determination of cholinesterase inhibitory activity of natural products [50,51,52]. In the cholinesterase inhibition assay of MBE (Figure 3B,C), both MBE and berberine showed the concentration-dependent inhibition activity of AChE and BChE, but the inhibition activity of berberine against AChE was significantly better than that against BChE, as reported in previous studies [51,53]. The inhibitory activity of MBE against BChE was significantly stronger than that against AchE. The IC_50_ of MBE was 101.82 ± 3.37 μg/mL (IC_50_ of positive berberine was 57.41 ± 0.21 μg/mL), whereas the IC_50_ of MBE for AChE was 179.47 ± 0.38 μg/mL (IC_50_ of positive berberine was 1.27 ± 0.03 μg/mL).

In the only study on the inhibition of the cholinesterase activity of MBE from *Morus alba* L., the IC_50_ values of MBE for AChE and BChE were, respectively, 46.78 ± 6.45 and 50.24 ± 3.01 μg/mL. The IC_50_ values of positive berberine for AChE and BChE were, respectively, 0.12 ± 0.00 and 9.45 ± 0.12 μg/mL [53]. The large difference in the IC_50_ values between the study and the previous report could possibly be ascribed to the differences in the reaction systems (e.g., final enzyme or substrate concentrations). However, the inhibition activities of berberine against AChE and BchE showed the similar trends. When the inhibition activities were calculated in terms of berberine equivalents, the MBE from Jialing 20 showed the significantly stronger BChE inhibitory activity than the MBE from *Morus alba* as well as better selectivity for BChE than AChE. Therefore, MBE might contain components with better BChE inhibitory activity.

The cholinesterase inhibitory components in mulberry branches have not been reported upon so far. Kuwanon G from mulberry roots was found to have better cholinesterase inhibitory activity, and its inhibitory activity against BChE was stronger than that against AchE [53]. UPLC-MS/MS analysis in this study showed that Kuwanon G was also contained in the MBE from Jialing 20 and might contribute to the inhibitory activity of MBE against BChE. In addition, the oxyresveratrol in MBE showed high inhibitory activity against BChE, with an IC_50_ value of 2.95 μM, but its inhibitory activity against AchE was low (IC_50_ > 400 μM) [54]. It was speculated that the inhibition activity of MBE against BchE might be ascribed to multiple components. Corresponding active ingredients and mechanism need to be further investigated.

### 3.5. Protection of MBE against ox-LDL-Induced HUVECs Damage

**Figure 4:**
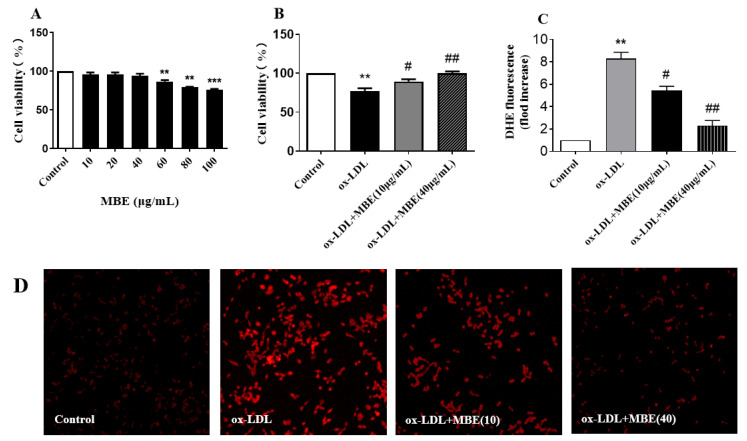
MBE ameliorated ox-LDL-induced damage in HUVECs. (**A**), Effect of MBE on the survival rate of HUVECs; (**B**), effect of MBE on the survival rate of ox-LDL-induced HUVECs; (**C**), relative content of ROS; (**D**), DHE staining of ROS. ** *p* < 0.01, versus control group; *** *p* < 0.001, versus control group; # *p* < 0.05, versus ox-LDL group; ## *p* < 0.01, versus ox-LDL group.

Atherosclerosis is a common cardiovascular disease, and its development process is closely related to the endothelial cell damage mechanism. ox-LDL can cause vascular endothelial cell damage and dysfunction through various pathways and can promote the development of atherosclerosis. The cytotoxicity results (Figure 4A) showed that MBE in the range of 10 to 40 μg/mL was not significantly toxic to HUVECs. Therefore, the highest concentration of MBE was subsequently selected to be 40 μg/mL in subsequent assays.

In the MBE protection assay against ox-LDL-induced HUVECs damage (Figure 4B), the activity of HUVECs exposed to ox-LDL was inhibited compared to those in the blank group. The survival rate of HUVECs exposed to 40 μg/mL MBE and ox-LDL was significantly higher than that of HUVECs exposed to ox-LDL (*p* < 0.01). Even 10 μg/mL MBE significantly increased the survival rate of the HUVECs (*p* < 0.05), indicating that MBE had a certain protective effect on ox-LDL-induced HUVECs damage. In other words, MBE has potential cardiovascular protective activity.

Reactive oxygen species can cause vascular endothelial cell dysfunction and vascular wall cell damage, which, in turn, develops into atherosclerosis. Removing excess ROS from the body may be one of the key points for the prevention and treatment of atherosclerosis [55]. DHE is a commonly used fluorescent detection probe for ROS since it can freely enter cells and can be dehydrogenated under the action of intracellular superoxide anions to form ethidium bromide, which can bind to DNA to produce red fluorescence under the excitation of ultraviolet rays. Within a certain range, the intracellular red fluorescence intensity indicated the concentration of superoxide anions. The red fluorescence intensity of HUVECs exposed to ox-LDL was significantly higher than that in the normal group (Figure 4D), suggesting the significantly elevated ROS level in HUVECs. In contrast, the intracellular ROS levels were significantly reduced after the HUVECs were treated with 10 μg/mL or 40 μg/mL MBE. In the processed results from Image J (Figure 4C), MBE effectively reduced the level of ROS in HUVECs under ox-LDL treatment and displayed potential preventive or therapeutic activity against atherosclerosis.

The activity of mulberry branch and its main components to protect ox-LDL-induced HUVECs damage has not been reported upon, but Kuwanon G was reported to reduce atherosclerosis by inhibiting the formation of foam cells and inflammatory response [56]. Kuwanon G might be the active component with protection against ox-LDL-induced HUVECs damage in MBE, but the active components in MBE and the corresponding mechanism remain to be further explored.

## 4. Conclusions

In this study, the chemical components of the MBE from Jialing 20 were analyzed and identified by UPLC-MS/MS for the first time. Then, the in vitro activities of antioxidation, α-glucosidase inhibition, cholinesterase inhibition, and protection against ox-LDL-induced HUVECs damage were determined. The MBE from Jialing 20 contained various active components such as flavonoids, polyphenols, and alkaloids, and displayed good antioxidant activity and promising applications in terms of hypoglycemic effect, Alzheimer’s disease inhibition, and cardiovascular protection. The study indicated that the MBE from Jialing 20 has potential as a food additive for the prevention of age-related diseases.

## Figures and Tables

**Table 1:** Tentatively identified compounds from Jialing 20 (*Morus multicaulis*) mulberry branch by UPLC-ESI-MS/MS.

No.	RT	Formula	m/z	MS/MS	Name	Adducts	Mass Error (ppm)	Raw Abundance	Fragmentation Score	Reference
1	0.57	C_6_H_13_NO_4_	164.0916	80.0493, 69.0331, 146.0814	1-Deoxynojirimycin	M+H	−0.67	546.60	92.3	TCM
2	1.93	C_16_H_18_O_9_	353.0880	191.0559, 179.0355	Neochlorogenic acid	M−H	0.47	216.73	82.3	TCM
3	2.21	C_15_H_16_O_9_	339.0718	177.0190, 133.0290	Esculin	M−H	−1.04	4822.19	85.2	TCM
		C_15_H_16_O_9_	341.0865	179.0331, 133.0274		M+H	−1.06	1025.21	90.6	TCM
4	2.49	C_26_H_32_O_14_	567.1732	243.0663, 405.1193	Mulberroside A	M−H	0.31	9166.15	93.0	TCM
		C_26_H_32_O_14_	569.1839	245.0798, 227.0692, 407.1312		M+H	−4.48	252.79	80.5	TCM
5	2.68	C_16_H_18_O_9_	353.0879	191.0575	Chlorogenic acid	M−H	0.14	850.31	94.5	TCM
6	2.8	C_15_H_16_O_8_	325.0916	163.0384	Skimmin	M+H	−0.75	58.25	99.7	TCM
7	2.85	C_16_H_18_O_9_	399.0928	191.0351, 176.0112,148.0174	Scopolin	M+FA−H	−1.29	722.21	94.0	TCM
		C_16_H_18_O_9_	355.1014	193.0491, 133.0272, 178.0245		M+H	−2.57	167.72	94.5	TCM
8	2.94	C_9_H_6_O_4_	177.0196	133.0287, 149.0240	7,8-Dihydroxycoumarin	M−H	1.36	310.70	86.8	TCM
9	2.97	C_7_H_6_O_4_	153.0193	67.0188, 109.0303, 65.0398	2,4-Dihydroxybenzoic acid	M−H	0.01	453.74	99.2	TCM
10	4.08	C_9_H_8_O_3_	163.0401	119.0502, 93.0345, 117.0332	2-Hydroxycinnamic acid	M−H	−0.09	101.52	94.1	TCM
11	4.44	C_9_H_6_O_3_	161.0243	133.0295	7-Hydroxycoumarin	M−H	−0.97	284.98	77.0	TCM
		C_9_H_6_O_3_	163.0391	77.0389, 107.0513		M+H	1.06	42.80	88.6	TCM
12	4.65	C_10_H_8_O_4_	191.0350	176.0117, 148.0172	6-Hydroxy-7-methoxycoumarin	M−H	0.17	218.22	82.7	TCM
13	4.66	C_10_H_8_O_4_	193.0502	178.0268, 133.0294, 150.0320	Scopoletin	M+H	1.70	646.40	78.7	TCM
14	4.83	C_20_H_22_O_8_	435.1292	227.0709, 185.0603, 389.1201	Polydatin	M+FA−H	−1.18	71.60	70.8	TCM
15	5.08	C_15_H_12_O_7_	303.0507	125.0241, 177.0193, 217.0508	3,5,7,3′,4′-Pentahydroxyflavanone	M−H	−1.01	8497.43	67.9	TCM
		C_15_H_12_O_7_	305.0659	153.0176, 123.0435		M+H	−0.95	847.04	77.0	TCM
16	5.14	C_21_H_20_O_12_	463.0869	301.0356, 151.0052	Spiraeoside	M−H	−2.78	180.28	89.9	TCM
17	5.16	C_21_H_20_O_12_	465.1012	303.0495, 257.0413	Hyperoside	M+H	−3.29	91.81	82.1	TCM
18	5.54	C_14_H_12_O_4_	243.0662	175.0760, 159.0448, 199.0762	Oxyresveratrol	M−H	−0.44	41,040.20	93.0	TCM
		C_14_H_12_O_4_	245.0811	107.0486, 161.0588, 181.0642		M+H	−0.19	12,078.74	81.1	TCM
19	5.94	C_21_H_22_O_10_	433.1139	271.0621, 119.0513, 365.0850	Naringenin-7-O-glucoside	M−H	−0.26	30.86	74.3	TCM
20	5.94	C_21_H_20_O_1_1	447.0929	285.0400, 257.0459	Kaempferol-7-O-β-D-glucopyranoside	M−H	−0.89	171.05	75.5	TCM
21	6.36	C_14_H_12_O_4_	243.0664	159.0458, 201.0558	Piceatannol	M−H	0.55	324.46	75.6	TCM
22	7.12	C_15_H_10_O_6_	285.0404	151.0048, 241.0509, 267.0360	Luteolin	M−H	−0.04	245.55	67.2	TCM
23	7.18	C_10_H_18_O_4_	201.1139	139.1137, 183.1048	Sebacic acid	M−H	3.21	27.21	95.5	TCM
24	7.27	C_10_H_10_O_3_	177.0561	117.0350, 145.0304	Methyl 4-hydroxycinnamate	M−H	2.30	19.37	78.2	TCM
25	7.33	C_14_H_12_O_3_	273.0770	227.0720, 185.0611, 209.0604	Resveratrol	M+FA−H	0.86	31.51	89.4	TCM
26	10.68	C_40_H_36_O_11_	693.2291	137.0227, 203.0701, 365.1010	Kuwanon G	M+H	−4.77	5434.08	68.5	TCM
		C_40_H_36_O_11_	691.2192	581.1812, 353.1023		M−H	0.98	10,494.42	90.2	TCM
27	11.06	C_18_H_34_O_4_	313.2385	183.1371, 295.2283	12,13-Dihydroxy-9Z-octadecenoic acid	M−H	0.29	58.35	92.7	TCM
28	11.13	C_25_H_26_O_6_	421.1635	299.1276, 309.0390	Mulberrin	M−H	0.17	16,531.24	-	[34]
29	11.32	C_18_H_39_NO_3_	318.2998	60.0440, 300.2894	Phytosphingosine	M+H, M+Na	−2.45	999.00	86.8	TCM
30	11.46	C_45_H_44_O_11_	759.2827	581.1841, 353.1017	Kuwanon H	M−H	0.28	3452.38	-	[35]
31	12.15	C_15_H_12_O	191.0854	165.0040	Chalcone	M+H−H_2_O	−0.81	128.30	84.2	TCM
32	12.41	C_18_H_32_O_3_	295.2277	277.2154, 183.1409	9(10)-Epoxy-12Z-octadecenoic acid	M−H	−0.55	2353.73	77.5	TCM
33	12.73	C_25_H_24_O_6_	419.1494	297.1126, 309.1122, 217.0504, 350.0788	Morusin	M−H	0.15	13,245.32	-	[36]
34	13.94	C_21_H_36_O_4_	353.2674	261.2201	Monolinolenin (9c,12c,15c)	M+H	−3.15	1235.93	91.6	TCM
35	14.9	C_21_H_38_O_4_	355.2844	263.2374, 245.2272, 337.2738	1-Monolinoleoyl-rac-glycerol	M+H	−1.28	1494.67	87.9	TCM
36	14.91	C_16_H_32_O_3_	271.2278	225.2246	2-Hydroxypalmitic acid	M−H	−0.13	169.52	82.8	TCM
37	15.47	C_19_H_38_O_4_	331.2840	57.0695, 313.2730, 239.2370	1-Palmitoylglycerol	M+H	−0.72	183.92	96.5	TCM
38	15.69	C_18_H_32_O_2_	325.2373	279.2323	Linoleic acid	M+FA−H	−3.96	147.72	82.8	TCM
39	15.7	C_18_H_32_O_2_	281.2474	55.0544, 69.0701, 83.0856, 97.1007	10E,12Z-Octadecadienoic acid	M+H	−0.55	1828.19	81.1	TCM
40	17.15	C_18_H_34_O	267.2676	67.0545, 81.0685, 95.0854, 109.0993	cis,cis-9,12-Octadecadien-1-ol	M+H	−2.38	163.02	84.2	TCM
41	17.5	C_19_H_36_O_3_	295.2624	263.2372, 245.2260, 55.0551, 69.0706, 81.0700, 95.0867,	Ricinoleic acid methyl ester	M+H−H_2_O	−2.48	378.47	93.0	TCM
42	18.78	C_29_H_50_O	397.3830	147.1182, 161.1323	*β*-Sitosterol	M+H−H_2_O	0.31	792.17	77.3	TCM

RT: retention time; RA: raw abundance; FA: formic acid, HCOOH. M+H, M+H−H_2_O, and M+Na are adduct ions in positive mode; M−H and M+FA−H are adduct ions in negative mode; - means the fragment is not compared with the TCM database; TCM is TCM_2.0_MSMS Library (AB Sciex).

## Data Availability

Not applicable.
